# Changing partners: transcription factors form different complexes on and off chromatin

**DOI:** 10.15252/msb.20145936

**Published:** 2015-01-21

**Authors:** Zongling Ji, Andrew D Sharrocks

**Affiliations:** Faculty of Life Sciences, University of ManchesterManchester, UK

## Abstract

The current knowledge on how protein–protein interactions regulate the function of transcription factors (TFs) has remained limited due to an incomplete knowledge of their interaction partners. In their recent work*,* Chen and colleagues (Li *et al*, 2015) analyse the interactome of 56 TFs and reveal distinct chromatin-associated and soluble TF complexes.

See also: X Li *et al* (January 2015)

The people we interact with shape the way we work, and these interactions change depending on where we are in the workplace. In a similar way, a protein's function is generally modulated by its interactions with other proteins, and these interactions can potentially change depending on the subcellular location of the protein. This is especially true for TFs that control gene expression largely by recruiting other proteins to chromatin. Discovering the interaction partners of TFs is therefore an important step towards understanding how they function to control gene expression and hence sculpt cellular phenotypes. In their recent study, Li *et al* (2015) address this important issue by systematically uncovering the interactome of 56 mammalian TFs. Importantly, they investigate the soluble and chromatin-associated cellular fractions and demonstrate that TFs bind to different partner repertoires when they are located on or off chromatin (Fig1). This study therefore provides a valuable resource for further investigation of TF function and regulation.

**Figure 1:**
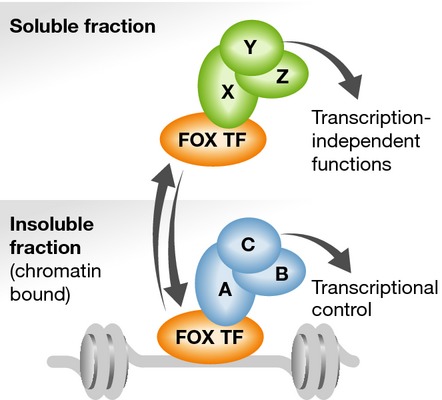
Transcription factors form different complexes on and off chromatin Two different types of complexes associate with Forkhead transcription factors (FOX TF) in the soluble (top; proteins X–Z) and insoluble (bottom; proteins A–C) cellular fractions. Complexes forming in the insoluble fraction are generally associated with transcriptional control and chromatin regulation, whereas those in the soluble fraction are associated with transcription-independent processes.

TFs control gene expression through interacting with the DNA embedded in chromatin. While this paradigm is well established, we are still far from being able to understand even how a single TF functions at a single promoter. Co-regulatory protein recruitment is a key aspect of TF function and it is becoming obvious that each TF can interact with a distinct repertoire of co-regulators. Over the years, we have built up a piecemeal understanding of TF interactions through the identification of binary interactions with a range of co-regulatory proteins. For example, over four hundred proteins have been shown to bind to the related histone acetyl transferases and transcriptional co-activators p300 and CBP including dozens of TFs (reviewed in Bedford *et al*, 2010). More recently, proteomic approaches have been applied to uncover the interactomes of mammalian TFs such as the oestrogen receptor using either a DNA-binding site-mediated precipitation approach (Foulds *et al*, 2013) or immunoprecipitation from cross-linked chromatin extracts (Mohammed *et al*, 2013). Importantly, the latter study was performed on chromatin, therefore more likely reflecting the function of the transcription factor in its natural context. Reciprocally, a large scale mass spectrometry (MS)-based study uncovered the “complexomes” of a large number of human transcriptional co-regulators (Malovannaya *et al*, 2011).

To begin to analyse the interactomes of mammalian TFs, Li *et al* (2015) first focus on 37 members of the Forkhead (FOX) TF family. They show that each of these TFs has a different interactome, which changes depending on whether the protein is isolated from the soluble cellular fraction or the chromatin-associated insoluble fraction. This is one of the major highlights of the work and illustrates that TFs change their interaction partners according to their subcellular localisation. This general phenomenon is further exemplified by studying an additional set of 19 transcription factors, which are representative members of a range of different families. Two FOX proteins are studied in further detail. FOXM1 is shown to interact with its anticipated partners within the B-Myb–MuvB (MMB) complex on chromatin (Sadasivam *et al*, 2012; Chen *et al*, 2013), but it is also shown to bind to novel partners, including several components of the tRNA splicing complex, in the soluble compartment. The latter observation suggests a potential novel regulatory role for this TF. Less is uncovered about FOXN2 on chromatin, but a co-associating protein degradation complex is identified in the soluble fraction, suggesting a route for FOXN2 degradation. Thus, the proteomic data sets generated in this study permit novel predictions for TF function to be postulated and taken forward for future investigation.

However, while clearly an important step forward, the current study does have limitations. For example, the subcellular fractionation to “soluble” and “insoluble” is rather crude, and more careful dissection of cellular compartments might prove to be more revealing. Like any proteomic study, the resulting interactomes only allow functional outcomes to be inferred and further functional investigations are required. More generally, the results also show an amalgamation of cellular states and hence lose dynamic information about changes that might occur on promoters and enhancers due to signalling pathway status or to different stages in the transcriptional process. For example, it is well established that FOXM1 functions through the MMB complex during the late G2 and early M phase of the cell cycle (Sadasivam *et al*, 2012), but it is unclear what happens to FOXM1 outside this time window. Finally, while this study clearly shows that within a given cell, a transcription factor can swap its partner proteins according to its subcellular localisation, it is likely that partner swapping will also occur in a cell type-dependent manner. Nevertheless, the current study does provide a useful starting point to begin to address many of these issues.

By building interaction maps for both TFs and transcriptional co-regulators, we move closer to understanding how TFs function to control gene regulation. Fundamentally, gaining insights into this process is critical for elucidating how cellular phenotypes are established and modified in response to internal and external signals. Perhaps more importantly, this knowledge will contribute to our understanding of disease states such as cancer where gene regulatory networks are dramatically altered and it will potentially help improve regenerative medicine interventions involving the rewiring of transcriptional regulatory networks.
